# Total antioxidant capacity is associated with mortality of patients with severe traumatic brain injury

**DOI:** 10.1186/s12883-015-0378-1

**Published:** 2015-07-25

**Authors:** Leonardo Lorente, María M. Martín, Teresa Almeida, Pedro Abreu-González, Luis Ramos, Mónica Argueso, Marta Riaño-Ruiz, Jordi Solé-Violán, Alejandro Jiménez

**Affiliations:** Intensive Care Unit, Hospital Universitario de Canarias, Ofra, s/n. La Laguna, 38320 Santa Cruz de Tenerife, Spain; Intensive Care Unit, Hospital Universitario Nuestra Señora de Candelaria, Crta del Rosario s/n, Santa Cruz de Tenerife, 38010 Spain; Unidad de Genética. Instituto de Enfermedades Tropicales y Salud Pública de Canarias, Universidad de La Laguna, Campus de Anchieta, Avda. Astrofísico Francisco Sánchez s/n, La Laguna, Tenerife 38071 Spain; Deparment of Phisiology. Faculty of Medicine, University of the La Laguna, Santa Cruz de Tenerife, Spain; Intensive Care Unit, Hospital General La Palma, Buenavista de Arriba s/n, Breña Alta, La Palma 38713 Spain; Intensive Care Unit, Hospital Clínico Universitario de Valencia, Avda. Blasco Ibáñez n°17-19, Valencia, 46004 Spain; Servicio de Bioquímica Clínica, Complejo Hospitalario Universitario Insular Materno-Infantil, Plaza Dr. Pasteur s/n, Las Palmas de Gran Canaria, 35016 Spain; Intensive Care Unit, Hospital Universitario Dr. Negrín, CIBERES, Barranco de la Ballena s/n, Las Palmas de Gran Canaria, 35010 Spain; Research Unit, Hospital Universitario de Canarias, Ofra, s/n. La Laguna, 38320 Santa Cruz de Tenerife, Spain

**Keywords:** Total antioxidant capacity, Brain trauma, Patients, Mortality, Injury

## Abstract

**Background:**

Previously, circulating total antioxidant capacity (TAC) in traumatic brain injury (TBI) patients has been scarcely studied and only in studies of small sample size (lower than 55 TBI patients). In one study were found higher serum TAC in non-survivor than in survivor TBI patients; however, an association between circulating TAC and mortality in patients with TBI has not been previously reported. Thus, the objective of this study was to determine whether there is an association between circulating TAC, peroxidation state and mortality in patients with severe TBI.

**Methods:**

This was a multicenter, observational and prospective study was carried out in six Spanish Intensive Care Units. We included patients with severe TBI defined as Glasgow Coma Scale (GCS) lower than 9. We excluded patients with Injury Severity Score (ISS) in non-cranial aspects higher than 9. We measured serum TAC on day 1 of TBI. The 30-day mortality was established as endpoint.

**Results:**

Non-surviving TBI patients (*N* = 27) showed higher serum TAC (*P* < 0.001) than survivor ones (*N* = 73). Logistic regression analyses showed that serum TAC higher than 2.59 nmol/mL were associated with 30-day mortality controlling for APACHE-II and CT classification (OR = 4.40; 95 % CI = 1.14–16.98; *P* = 0.03), controlling for GCS and age (OR = 5.88; 95 % CI = 1.57–22.06; *P* = 0.009), and controlling for CT classification and admission abnormal pupils (OR = 3.89; 95 % CI = 1.30–11.61; *P* = 0.02). There was an association between serum TAC and malondialdehyde (a biomarker of lipid peroxidation) levels (rho = 0.25; *p* = 0.01), APACHE-II score (rho = 0.23; *p* = 0.03) and GCS (rho = −0.21; *p* = 0.04).

**Conclusions:**

To our knowledge, our series is the largest reporting data on circulating TAC in patients with severe TBI. The most relevant and new findings of our study were that there is an association between circulating TAC and peroxidation state and mortality in patients with severe TBI.

## Background

Traumatic brain injury (TBI) could leads to death, disability, and high resources consume [1]. TBI causes two kinds of injury in the brain: 1) Primary injury, which appears at moment of the impact and is due to the initial physical forces applied to brain; 2) Secondary injury, which appears in the following hours or days, and leads to neuroinflammatory response and free radical generation [2].

Reactive oxygen species (ROS) are produced after TBI and they are involved in the secondary brain injury [3–6]. Under physiologic conditions, ROS are carefully balanced by the action of antioxidant defenses in cerebral tissue. These antioxidants do not work alone but they establish complex interactions with each others [7]. Thus, measurement of total antioxidant capacity (TAC) in serum or plasma, may give more biologically relevant information about patient antioxidant status than that obtained from measuring concentrations of individual compounds [8]. However, when antioxidant defenses are overwhelmed, oxidative stress results, which can cause significant damage to lipids, proteins, carbohydrates and nucleic acids [9].

Previously, there was found that non-survivor TBI patient showed higher lipid peroxidation than survivor patients [10–13]; however, there was also found the absence of that association [14]. In addition, circulating TAC in TBI patients has been scarcely studied and only in studies of small sample size (lower than 55 TBI patients) [15–17]. In one study were found higher serum TAC in non-survivor than in survivor TBI patients [15]; however, an association between circulating TAC and mortality in patients with TBI has not been previously reported. Thus, the objective of this study was to determine whether there is an association between circulating TAC, peroxidation state and mortality in patients with severe TBI.

## Methods

### Design and subjects

A prospective, observational, multicenter study was carried out between 2009–2012 in six Spanish Intensive Care Units. The study was approved by the Institutional Review Board of the 6 participating hospitals: Hospital Universitario de Canarias (La Laguna), Hospital Universitario Nuestra Señora de Candelaria (Santa Cruz de Tenerife), Hospital Clínico Universitario de Valencia (Valencia), Hospital General de La Palma (La Palma), Hospital Universitario Dr. Negrín (Las Palmas de Gran Canaria), Hospital Insular (Las Palmas de Gran Canaria). Written informed consent from the legal guardians of the patients was obtained.

The patient cohort, including 100 patients with severe TBI, is the same that was used in a previous publication by our team [13]. Previously we determined malondialdehyde (MDA) to assess lipid peroxidation, and at current study we have analyzed TAC.

We included patients with severe TBI defined as Glasgow Coma Scale (GCS) lower than 9 points [18]. We excluded patients with age less than 18 years, pregnancy, inflammatory or malignant disease and Injury Severity Score (ISS) in non-cranial aspects higher than 9 points [19].

### Variables recorded

The following variables were recorded for each patient at hospital admission: sex, age, brain lesion according to the Marshall computer tomography (CT) classification [20], ISS, GCS, lactic acid, platelets, international normalized ratio (INR), activated partial thromboplastin time (aPTT), fibrinogen, and Acute Physiology and Chronic Health Evaluation II (APACHE II) score [21].

### End-point

The 30-day mortality was established as endpoint.

### Blood sample collection

Blood samples were collected on day 1 of TBI in tubes with separator gel. After coagulation during 10 min at room temperature, serum was obtained by centrifugation at 1000 g for 15 min. The samples were aliquoted and frozen at −80 °C until determination.

### Serum TAC analysis

TAC in serum samples was evaluated using antioxidant assay kit (Cayman Chemical Corporation, Ann Arbor, USA). The assay relies on the ability of antioxidants in the sample to inhibit the oxidation of ABTS® (2,2'-azino-di-[3-ethylbenzthiazoline sulphonate]) to ABTS®^+^ by metmyoglobin. The capacity of the antioxidants in the sample to prevent ABTS oxidation, is compared with that of Trolox, a water-soluble tocopherol analogue, and is quantified as molar Trolox equivalents. All samples were assayed in duplicate at 20-fold dilutions in assay buffer following manufacturer’s instructions. Absorbance at 750 nm was measured using the EnSpire multimode plate reader (PerkinElmer,Waltham, MA, USA). The serum concentration of TAC was expressed in mmol/L. The detection limit of this assay was 0.04 mmol/L; the intra- and inter-assay CV were 3.4 % and 3.0 %, respectively. To avoid the possible dispersion of serum TAC results, all the samples were processed at the same time, at the end of the recruitment process. Serum TAC determination was performed by a laboratory technician blinded to all clinical data. TAC assay was performed in the Genetic Unit of the Instituto de Enfermedades Tropicales y Salud Pública de Canarias of the University of the La Laguna (Tenerife, Spain).

### Serum MDA analysis

Malondialdehyde (MDA) is an end-product formed during this lipid peroxidation, due to degradation of cellular membrane phospholipids. MDA is released into extracellular space and finally into the blood; and has been used as an effective biomarker of lipid oxidation [22, 23].

Serum MDA levels were measured using thiobarbituric acid-reactive substance (TBARS) method as described by Kikugawa et al. [24]. The pink complex of samples was extracted in n-butanol. Each sample was placed in a 96-well plate and read at 535 nm in a microplate spectrophotometer reader (Benchmark Plus, Bio-Rad, Hercules, CA, USA). The detection limit of this assay was 0.079 nmol/ml; the intra- and inter-assay CV were 1.82 % and 4.01 %, respectively. The serum concentration of MDA was expressed in nmol/ml. To avoid the possible dispersion of serum MDA level results, all the samples were processed at the same time, at the end of the recruitment process. MDA determination was performed by a laboratory technician blinded to all clinical data. The assay of MDA levels was centralized in the Department of Physiology, Faculty of Medicine (University of the La Laguna. Santa Cruz de Tenerife. Spain).

### Statistical methods

Categorical variables are reported as frequencies and percentages; and comparisons between groups were carried out with chi-square test. Continuous variables are reported as medians and interquartile ranges; and comparisons between groups were carried out using Wilcoxon-Mann–Whitney test.

Receiver operating characteristic (ROC) analysis was carried out to determine the goodness-of-fit of serum TAC to predict 30-day mortality. Kaplan-Meier analysis of survival at 30 days and comparisons by log-rank test were carried out using serum TAC lower/higher than 2.59 nmol/mL as the independent variable and survival at 30 days as the dependent variable.

Multiple binomial logistic regression analyses were carried out to determine the association between serum TAC and 30-day mortality. We constructed three multiple binomial logistic regression models with only three predictor variables in each model, due to that the number of events (death) was 27, to avoid a final model of order slightly higher than required due to an over fitting effect. In the first model were included CT with high risk of death (types 3,4,6), APACHE-II score and serum TAC > 2.59 nmol/mL. In the second model were included age, GCS and serum TAC > 2.59 nmol/mL. In the third model were included CT with high risk of death (types 3,4,6), admission abnormal pupils and serum TAC > 2.59 nmol/mL. Odds Ratio and 95 % confidence intervals were calculated as measurement of the clinical impact of the predictor variables. We calculated for each multiple binomial logistic regression analysis, the overall predictive capacity to predict 30-day mortality and its predictive capacity for 30-day mortality (including area under curve, sensitivity, specificity, positive likelihood ratio, negative likelihood ratio, positive predicted value, negative predicted value).

A *P* value of less than 0.05 was considered statistically significant. Statistical analyses were performed with SPSS 17.0 (SPSS Inc., Chicago, IL, USA) and NCSS 2000 (Kaysville, Utah) and LogXact 4.1, (Cytel Co., Cambridge, MA).

## Results

Comparisons between surviving (*N* = 73) and non-surviving (*N* = 27) patients are shown in Table 1. Non-surviving TBI patients showed higher rate of female (*p* = 0.02). There were statistically significant differences in CT classification between non-surviving and surviving patients (*p* = 0.002). Non-surviving TBI patients showed lower GCS (*p* < 0.001), and higher age (*p* < 0.001) and APACHE-II score (*p* < 0.001) than survivor ones. In addition, non-surviving patients showed higher serum TAC (*p* < 0.001) and MDA (*p* < 0.001) levels than surviving ones.

**Table 1:** Comparison between survivor and non-survivor TBI patients

	Non-survivors	Survivors	P-value
	(*n* = 27)	(*n* = 73)	
Sex female - n (%)	11 (40.7)	12 (16.4)	0.02
Admission abnormal pupils - n (%)	12 (44.4)	11 (15.1)	0.003
Marshall CT classification - n (%)			0.002
Non-evacuated mass lesion	8 (29.6)	3 (4.1)	
Evacuated mass lesion	5 (18.5)	26 (35.6)	
Type 4 injury	6 (22.2)	10 (13.7)	
Type 3 injury	5 (18.5)	13 (17.8)	
Type 2 injury	3 (11.1)	21 (28.8)	
Type 1 injury	0	0	
CT with high risk of death (types 3,4,6) - n (%)	19 (70.4)	26 (35.6 %)	0.003
Age (years) - median (p 25–75)	66 (45–76)	47 (32–67)	<0.001
Temperature (°C) - median (p 25–75)	36.0 (35.0–37.0)	37. (35.6–37.3)	0.12
Sodium (mEq/L) - median (p 25–75)	141 (135–149)	139 (138–142)	0.19
Glycemia (g/dL) - median (p 25–75)	161 (142–189)	139 (120–163)	0.08
Leukocytes *10^3^/mm^3^ - median (p 25–75)	18.3 (10.7–23.9)	14.7 (10.2–19.3)	0.46
PaO2 (mmHg) - median (p 25–75)	141 (104–186)	151 (116–217)	0.34
PaO2/FI0_2_ ratio - median (p 25–75)	190 (154–316)	336 (242–407)	0.11
Bilirubin (mg/dl) - median (p 25–75)	0.75 (0.53–1.05)	0.50 (0.40–0.87)	0.045
Creatinine (mg/dl) - median (p 25–75)	0.95 (0.70–1.10)	0.80 (0.70–0.90)	0.44
Hemoglobin (g/dL) - median (p 25–75)	11.1 (9.4–12.3)	11.4 (10.4–13.0)	0.87
Glasgow Coma Scale score - median (p 25–75)	3 (3–6)	7 (6–8)	<0.001
Lactic acid (mmol/L) - median (p 25–75)	1.90 (1.15–4.55)	1.70 (1.23–2.50)	0.16
Platelets *10^3^/mm^3^ - median (p 25–75)	215 (139–264)	182 (143–252)	0.48
International normalized ratio - median (p 25–75)	1.22 (1.01–1.67)	1.03 (0.92–1.15)	0.15
aPTT (seconds) - median (p 25–75)	26 (25–31)	28 (25–32)	0.86
Fibrinogen (mg/dl) - median (p 25–75)	376 (246–560)	350 (282–444)	0.32
APACHE-II score - median (p 25–75)	26 (25–32)	19 (17–23)	<0.001
Injury Severity Score - median (p 25–75)	25 (25–27)	25 (25–32)	0.24
Intracranial pressure (mmHg) - median (p 25–75)	20 (12–30)	15 (14–20)	0.27
Cerebral perfusion pressure (mmHg) - median (p 25-75)	60 (54–69)	68 (57–70)	0.46
Malondialdehyde (nmol/mL) - median (p 25–75)	1.99 (1.31–2.76)	1.35 (1.02–1.79)	<0.001
Total antioxidant capacity (nmol/mL) - median (p 25–75)	5.09 (2.78–9.95)	2.31 (1.85–2.84)	<0.001

*CT* Computer tomography, *p* percentile, *PaO*_*2*_ pressure of arterial oxygen, *FIO*_*2*_ fraction inspired oxygen, *aPTT* activated partial thromboplastin time, *APACHE II* Acute Physiology and Chronic Health Evaluation

* multiplied by

**Fig. 1:**
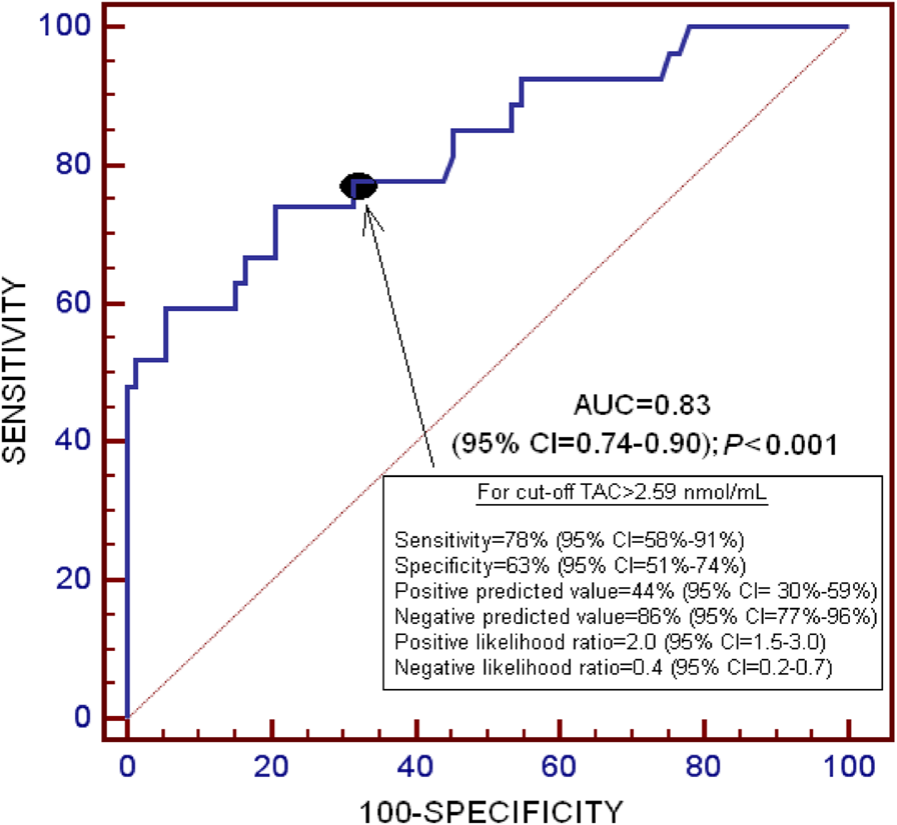
Receiver operation characteristic analysis using serum total antioxidant capacity (TAC) as predictor of mortality at 30 days

The area under the curve (AUC) for serum TAC as predictor of 30-day mortality was 0.83 (95 % CI = 0.74-0.90; *P* < 0.001) (Fig. 1).

Proportions of non-survivors was higher in patients with serum TAC higher than 2.59 nmol/mL (21of 48; 43.8 %) compared with those with lower serum TAC (6 of 52; 11.5 %). For this comparison power analysis was higher than 95 %.

**Fig. 2:**
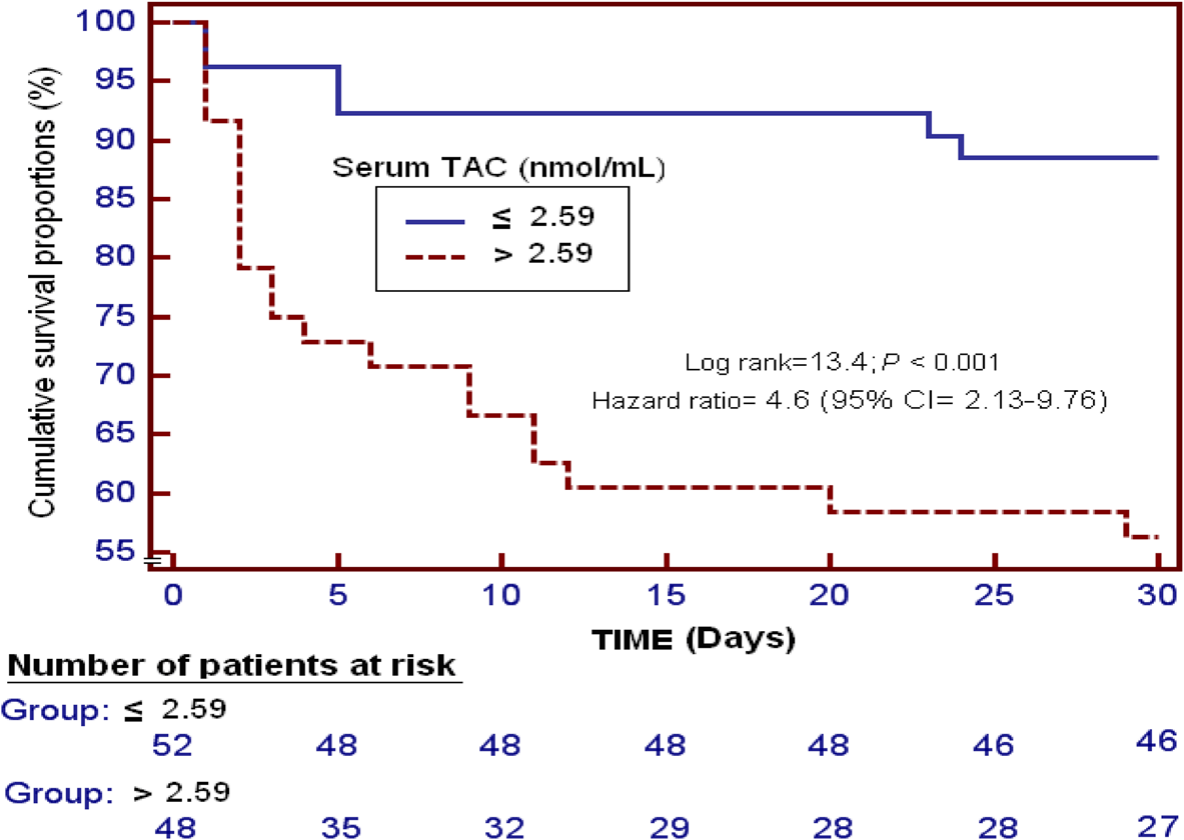
Survival curve at 30 days using 2.59 nmol/mL of serum total antioxidant capacity (TAC) as cut-off

Survival analysis showed that patients with serum TAC higher than 2.59 nmol/mL presented higher 30-day mortality than patients with lower levels (Hazard ratio = 4.6; 95 % CI = 2.13–9.76; *P* < 0.001) (Fig. 2).

Multiple binomial logistic regression analysis showed that serum TAC higher than 2.59 nmol/mL were associated with 30-day mortality controlling for APACHE-II and CT classification (OR = 4.40; 95 % CI = 1.14–16.98; *P* = 0.03), controlling for GCS and age (OR = 5.88; 95 % CI = 1.57–22.06; *P* = 0.009), and controlling for CT classification and admission abnormal pupils (OR = 3.89; 95 % CI = 1.30–11.61; *P* = 0.02) (Table 2). The overall predictive capacity for the first, second and third multiple binomial logistic regression analyses to predict 30-day mortality was 85.4 %, 82.3 % and 76.0 %, respectively. The area under curve for the first, second and third multiple binomial logistic regression analyses to predict 30-day mortality was 0.79, 0.74 and 0.59, respectively (Table 3).

**Table 2:** Multiple binomial logistic regression analysis to predict 30-day mortality

Variable	Odds ratio	95 % confidence interval	*P*
First model			
CT with high risk of death (types 3,4,6)	5.22	1.264–21.555	0.02
APACHE-II score	1.14	1.206–1.655	<0.001
Serum TAC > 2.59 nmol/mL	4.40	1.142–16.977	0.03
Second model			
Age	1.08	1.033–1.118	<0.001
GCS score	0.54	0.386–0.752	<0.001
Serum TAC > 2.59 nmol/mL	5.88	1.565–22.064	0.009
Third model			
CT with high risk of death (types 3,4,6)	3.84	1.317–11.202	0.01
Admission abnormal pupils	4.06	1.311–12.596	0.02
Serum TAC > 2.59 nmol/mL	3.89	1.301–11.607	0.02

*CT* Computer tomography, *APACHE II* Acute Physiology and Chronic Health Evaluation, *TAC* Total antioxidant capacity, *GCS* Glasgow Coma Scale

**Table 3:** Predictive capacity of the three multiple binomial logistic regression analysis to predict 30-day mortality

	First model	Second model	Third model
Overall predictive capacity	85.4 %	82.3 %	76.0 %
Area under curve; p-value	0.79; *p* < 0.001	0.74; *p* < 0.001	0.59; *p* = 0.03
Sensitivity (95 % CI)	64 (43–82)	56 (35–76)	22 (9–42)
Specificity (95 % CI)	93 (84–98)	92 (83–97)	96 (89–99)
Positive likelihood ratio (95 % CI)	9.1 (3.7–22.2)	6.6 (2.9–15.4)	5.4 (1.5–20.1)
Negative likelihood ratio (95 % CI)	0.4 (0.2–0.7)	0.5 (0.3–0.8)	0.8 (0.7–1.0)
Positive predicted value (95 % CI)	76 (53–92)	70 (46–88)	67 (30–93)
Negative predicted value (95 % CI)	88 (78–94)	86 (76–93)	77 (67–85)

*CI* confidence interval

There was an association between serum TAC and MDA levels (rho = 0.25; *p* = 0.01), APACHE-II score (rho = 0.23; *p* = 0.03) and GCS (rho = −0.21; *p* = 0.04).

## Discussion

To our knowledge, our series is the largest reporting data on circulating TAC in patients with severe TBI. The most relevant and new findings of our study were that there is an association between circulating TAC and peroxidation state and mortality in patients with severe TBI.

We found that non-survivor TBI patients showed higher serum TAC than survivor ones. These findings are in consonance with those of other previous study of small sample size [15]. In addition, these findings are consistent with the results of other studies in septic patients showing higher circulating TAC in non-survivor septic patients than in survivor ones [25–27]. A new aspect of our study was the association between serum TAC and mortality in severe TBI patients according to the results of regression analysis. That finding is also in consonance with the results of other study in septic patients showing that serum TAC is associated with mortality in severe septic patients [27]. Besides, we report for the first time that serum TAC could be used to predict mortality in patients with severe TBI according the results of ROC curve analysis.

We found a higher rate of death in female than in male. Previously, there was found a influence of the sex in the death rate [28, 29]. However, there was found a worse outcome in females [28], and in males [29]. And the influence of the hormones was pointed as the possible explanation for those findings in both cases; thus the influence and the cause of sex in TBI outcome remains unclear.

Another interesting finding of our study was the association between serum TAC and TBI severity, assessed by GCS. That finding is in consonance with that of other previous study of small sample size [16].

We found that non-survivor TBI patients showed higher serum MDA levels (as biomarker of lipid peroxidation) than survivor ones. That finding is in consonance with the results of other studies showing that non-survivor TBI patient had higher lipid peroxidation than survivor patients [10–13]; however, in other series was not found that association [14].

In addition, other novel aspect of our study was that there was a positive association between serum TAC and lipid peroxidation, assessed by serum MDA levels, in severe TBI patients.

We believe that all these findings might suggest that non-surviving TBI patients with respect to survivor ones have an increased production of ROS and the increased TAC attempts to compensate the high production of oxidant products to maintain the balance between oxidant and antioxidant state; however, in non-surviving TBI patients this increased TAC is not enough to compensate the high production of oxidants species and finally present a high ROS production and high peroxidation of lipids, proteins, carbohydrates and nucleic acids. From a therapeutic perspective, the use of antioxidant agents could be used as a new class of drugs for the treatment of patients with severe TBI according the results of animal models [30–34].

Finally, some limitations of our study should be recognized. First, the measure of other compounds of oxidant and antioxidant states would be desirable in order to better evaluate this balance. Second, we did not perform the analysis of serum TAC during follow-up. Third, we did not report data on many patients were excluded from the study and the motivation for missing. Fourth, blood samples for serum TAC were collected on the day of TBI; however, the timing of the blood sampling may be different among patients due to that the exact moment of the blood sampling was not reported. Thus, additional studies are needed to confirm the finding of our study.

## Conclusions

To our knowledge, our series is the largest reporting data on circulating TAC in patients with severe TBI. The most relevant and new findings of our study were that there is an association between circulating TAC and peroxidation state and mortality in patients with severe TBI.

## Abbreviations

TBI: Traumatic brain injury
TAC: Total antioxidant capacity
GCS: Glasgow Coma Scale
ISS: Injury Severity Score
PaO_2_: Pressure of arterial oxygen
FIO_2_: Fraction inspired oxygen
aPTT: activated partial thromboplastin time
APACHE II: Acute Physiology and Chronic Health Evaluation
